# Assessment of c-Kit immunohistochemical expression in cutaneous basal and squamous cell carcinomas

**DOI:** 10.1038/s41598-026-64048-z

**Published:** 2026-08-06

**Authors:** Doaa Nasr Eldin Sedky Haroun, Eman Ahmed Abd Elmaogod, Abla Sayed Mahmoud

**Affiliations:** Pathology department, Faculty of Medicine, Beni-Suef University, Beni-Suef, Egypt

**Keywords:** BCC, CSCC, C-Kit, Immunohistochemistry, Cancer, Oncology

## Abstract

Basal cell carcinoma (BCC) and cutaneous squamous cell carcinoma (cSCC) are the most prevalent cutaneous malignancies. C-Kit plays a role in tumor initiation, progress, recurrence, and metastasis. Several medicinal treatments target the c-Kit pathway. The aim of the study was to assess the expression of c-Kit in BCC and cSCC. Furthermore, the study aimed to correlate c-Kit expression with available pathological data of the tumor and clinical data of the patients. A total of 62 excision specimens from patients with BCC (n = **41)** and cSCC (n = **21)** were histopathologically and immunohistochemically evaluated using anti-c-Kit, a rabbit monoclonal antibody clone IHC526. More than half of BCC cases (63.4%) showed positive c-Kit expression. C-Kit expression was significantly correlated with age (p-value = 0.011). The intensity of positive c-Kit staining was statistically related to tumor size (p-value = 0.028). All cSCC cases (100%) showed negative c-Kit expression. Receiver operating characteristic (ROC) curve analysis demonstrated an area under the curve (AUC) of 0.817 (95% confidence interval: 0.714–0.920, p-value < 0.001), with a sensitivity of 63.4% and a specificity of 100% in differentiating BCC from cSCC. Assessment of c-Kit expression may be beneficial in the diagnosis of BCC and in distinguishing it from cSCC. Its high specificity supports its diagnostic utility; however, due to its moderate sensitivity, it should be interpreted in conjunction with routine histopathological findings. While this study was a diagnostic, it suggested that c-Kit inhibitors may have therapeutic effects in BCC cases that express c-Kit; however, more research is needed to validate this.

## Introduction

Basal and squamous cell carcinoma account for approximately 98% of cutaneous malignancies. They are the most prevalent cancers affecting people of white skin color^1^.

Based on the population of Gharbia, the National Cancer Registry in Egypt reported that the incidence of BCC and cSCC was 3.1% of all cancers in both sexes. This data, which spans nine years, was gathered retrospectively from 40,477 patients at Tanta Cancer Center to create Egypt’s largest population-based cancer registry^2^.

Ultraviolet radiation (UVR) seems to be the main etiological factor of BCC. Other risk factors include immunosuppression, aging, male sex, genetic predisposition, and fair skin^3^.

After BCC, cSCC is the second most prevalent non-melanoma skin cancer. Approximately one million instances of cSCC occur in the US each year. In the last three decades, the number of cSCCs has risen from 50% to 300%, and by 2030, the incidence in Europe will be double that of today^4^. With the exception of melanoma, cSCC is responsible for 75% of all skin cancer deaths^5^.

cSCC develops mostly in the eighth decade of life and is more common in males than in females. Furthermore, white populations with blue eyes and blonde or red hair have shown a high incidence rate. The risk to develop cSCC is affected by the lifelong exposure to UVR^6^.

The proto-oncogene c-KIT, a tyrosine kinase receptor protein, is encoded by the human KIT gene and is predominantly expressed in melanocytes, mastocytes, and germinal cells. The c-Kit receptor is involved in cellular proliferation, differentiation, and anti-apoptotic properties. It contributes to tumor occurrence, development, metastasis, and recurrence since it is expressed in certain malignancies^7^.

Previous studies have also evaluated c-Kit expression in BCC and cSCC, with variable results. Terada et al.^8^ reported high expression in BCC, with positivity in 93% of 66 cases. In contrast, Goto et al.^9^ demonstrated lower expression in cSCC, with positivity in 19.4% of 31 cases.

Several medicinal treatments target the c-Kit pathway. It is obvious that evaluating c-Kit expression in patients with BCC and cSCC can have therapeutic and diagnostic effects^10^.

## Methods

A total of 62 excision specimens of patients with BCC (n = **41)** and cSCC (n = **21)** were received at the Pathology department, Beni-Suef University Hospital during the period from September 2022 to September 2024. All lesions included in the present study were located on the face, which is chronically exposed to UVR. BCCs were classified into low-risk and high-risk groups according to their histopathological subtype. Low-risk tumors consisted of nodular BCC only, while high-risk tumors included micronodular, morpheaform, and basosquamous subtypes.

The study included archival formalin-fixed paraffin-embedded (FFPE) tissue blocks from 62 cases. Available well-established pathological and clinical data as age and sex were included.

### Aim of the study

- Evaluating c-Kit expression in BCC and cSCC for diagnostic purposes and suggesting future therapeutic implications.
- Correlation between c-Kit expression and tumor pathological data (such as tumor size, histopathological type, and grade) and clinical data of the patients (such as age and sex).

### Study design

This study is designed as a cross-sectional analytical study.

### Sample size

The sample size equation n = [DEFF × N × p × (1 − p)]/{ [d²/Z(1 − α/2)² × (*N* − 1)] + p × (1 − p) } was used in OpenEpi, Version 3.

At the 90% confidence level, the total sample size is 52 patients.

### Histopathological evaluation

Tumor paraffin blocks were sectioned at 4 μm thickness using a rotary microtome. For pathological re-evaluation, sections were stained with routine hematoxylin and eosin (H&E).

### Immunohistochemical evaluation

Tumor sections were mounted on positively charged slides. Immunohistochemistry (IHC) was performed on formalin-fixed paraffin-embedded (FFPE) tissue sections. Sections were deparaffinized in xylene and rehydrated through graded alcohols. Antigen retrieval was performed using microwave heating in citrate buffer (pH 6.0). Endogenous peroxidase activity was blocked using 3% hydrogen peroxide. The sections were then incubated with a ready-to-use anti-c-Kit rabbit monoclonal antibody (clone IHC526, GenomeMe, Cat# IHC000, Richmond, BC, Canada). Immunostaining was performed using the Dako Autostainer Link 48 system. Detection was performed using 3,3′-diaminobenzidine (DAB) as chromogen, followed by hematoxylin counterstaining. Slides were dehydrated, cleared, and mounted with DPX (11 and 12).

### Positive control

Parallel positive sections of gastrointestinal stromal tumor (GIST) cases were used as external positive controls for each set of slides.

### Interpretation of c-Kit immunostaining

C-Kit expression was identified by brownish staining in the cell membrane and/or cytoplasm of tumor cells. Expression was classified as follows:

negative: <1% of tumor cells, rarely positive: 1–4%, focally positive: 5–29%, and diffusely positive: 30–100%^9^.

Staining intensity was graded as follows:

weak: visible only under high-power view; moderate: easily visible under low-power view, but weaker than normal eccrine ducts staining; and strong: equal to or stronger than the normal eccrine ducts staining. In cases with heterogeneous intensity, the predominant grade was recorded^13^.

We considered negative or rare positive expression as negative and considered focally or diffusely positive expression as positive. Staining extent and staining intensity were assessed independently and recorded as separate parameters. No composite scoring system was used.

Pigmented BCCs were not represented in our study; therefore, melanin pigmentation did not interfere with the interpretation of c-Kit immunostaining.

### Examination and imaging of the slides

An Olympus BX53 light microscope was used to examine the slides, and Leica digital pathology slide scanners (Aperio LV1 IVD) were used to take images.

### Statistical analysis

The Statistical Package for the Social Sciences version 25 for Windows (IBM Corp., released 2017, Armonk, NY, USA) was used to code, enter, and analyze the gathered data.

First, descriptive statistics on the demographic and pathological data of the cases were analyzed.

Age and tumor size were recorded as mean and standard deviation (mean ± SD) and classified into two categories. All categorical data was expressed as count and percent. The chi-square test was used to assess associations between categorical variables. A p-value of 0.05 or less was considered significant. ROC curve analysis was performed to evaluate the diagnostic performance of c-Kit expression in differentiating BCC from cSCC. The AUC was calculated, and sensitivity and specificity were determined.

### Ethics approval and consent to participate

This study was performed in line with the principles of the Declaration of Helsinki. Approval was granted by the Ethics Committee of Beni-Suef University, December 2022, Approval number: FMBSUREC/06122022/Haroun.

Due to the retrospective nature of the study, the Ethics Committee of Beni-Suef University waived the need for obtaining informed consent. For information confidentiality and to ensure that it is only utilized for research, all patient-identifying information, including names, had been removed from the archival formalin-fixed paraffin-embedded (FFPE) tissue blocks and replaced with numbers.

## Results

Clinicopathological data and c-Kit expression of the studied cases of BCC in our study are demonstrated in Table 1. More than half of the participants were > 60 years old (61%). The ratio of males to females was 1.4:1, with males making up the majority (58.5%). Fifty-eight and a half percent of tumors measured ≤ 2 cm. About the histopathological variant, the studied cases were divided into low risk and high risk. More than half of the cases (70.7%) were low-risk tumors, consisting of nodular basal cell carcinoma (*n* = 29). The remaining cases were high-risk subtypes (29.3%), including basosquamous (*n* = 5), morpheaform (*n* = 5), and micronodular (*n* = 2). This study demonstrated that more than half of cases (63.4%) showed positive c-Kit expression. Our study showed that the expression of positive c-Kit staining was mainly diffuse (65.4%), and the intensity was mainly strong (73.1%).

**Table 1:** Clinicopathological data and c-Kit expression of the studied cases of BCC.

		Count	%
Histopathological diagnosis	Basal Cell Carcinoma	41	100.0%
Age	Mean ± SD	64 ± 12	
	Range	34–90 years	
	≤ 60	16	39.0%
	> 60	25	61.0%
Sex	Male	24	58.5%
	Female	17	41.5%
Size (cm)	Mean ± SD	2 ± 1.1	
	Range	0.6–4.7 cm	
	≤ 2	24	58.5%
	> 2	17	41.5%
Histopathological variants of BCC	Low risk	29	70.7%
	High risk	12	29.3%
C-Kit expression	Positive	26	63.4%
	Negative	15	36.6%
Expression of positive c-Kit staining	Focal	9	34.6%
	Diffuse	17	65.4%
Intensity of positive c-Kit staining	Weak	0	0%
	Moderate	7	26.9%
	Strong	19	73.1%

### C-Kit expression among studied cases

**Fig. 1:**
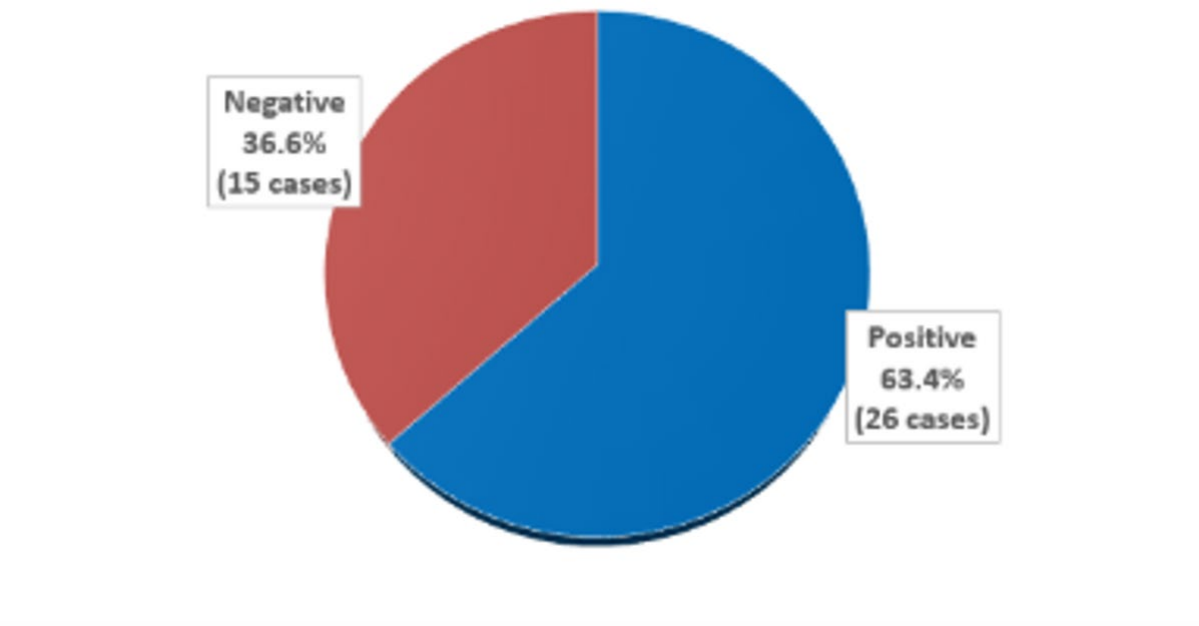
C-Kit expression in BCC.

This study demonstrated that more than half of cases (63.4%) showed positive c-Kit expression (Figure 1).

**Table 2:** Relation between expression of positive c-Kit staining and age, sex, tumor size, and histopathological variants.

		Expression of positive c-Kit staining				p-value
		Focal		Diffuse		
		Count	%	Count	%	
Age	≤ 60	1	11.1%	12	70.6%	0.011
	> 60	8	88.9%	5	29.4%	
Sex	Male	6	66.7%	7	41.2%	0.411
	Female	3	33.3%	10	58.8%	
Tumor Size (cm)	≤ 2	4	44.4%	13	76.5%	0.194
	> 2	5	55.6%	4	23.5%	
Histopathological variants	Low risk	6	66.7%	12	70.6%	1
	High risk	3	33.3%	5	29.4%	
	Total	9	100%	17	100%	

**Fig. 2:**
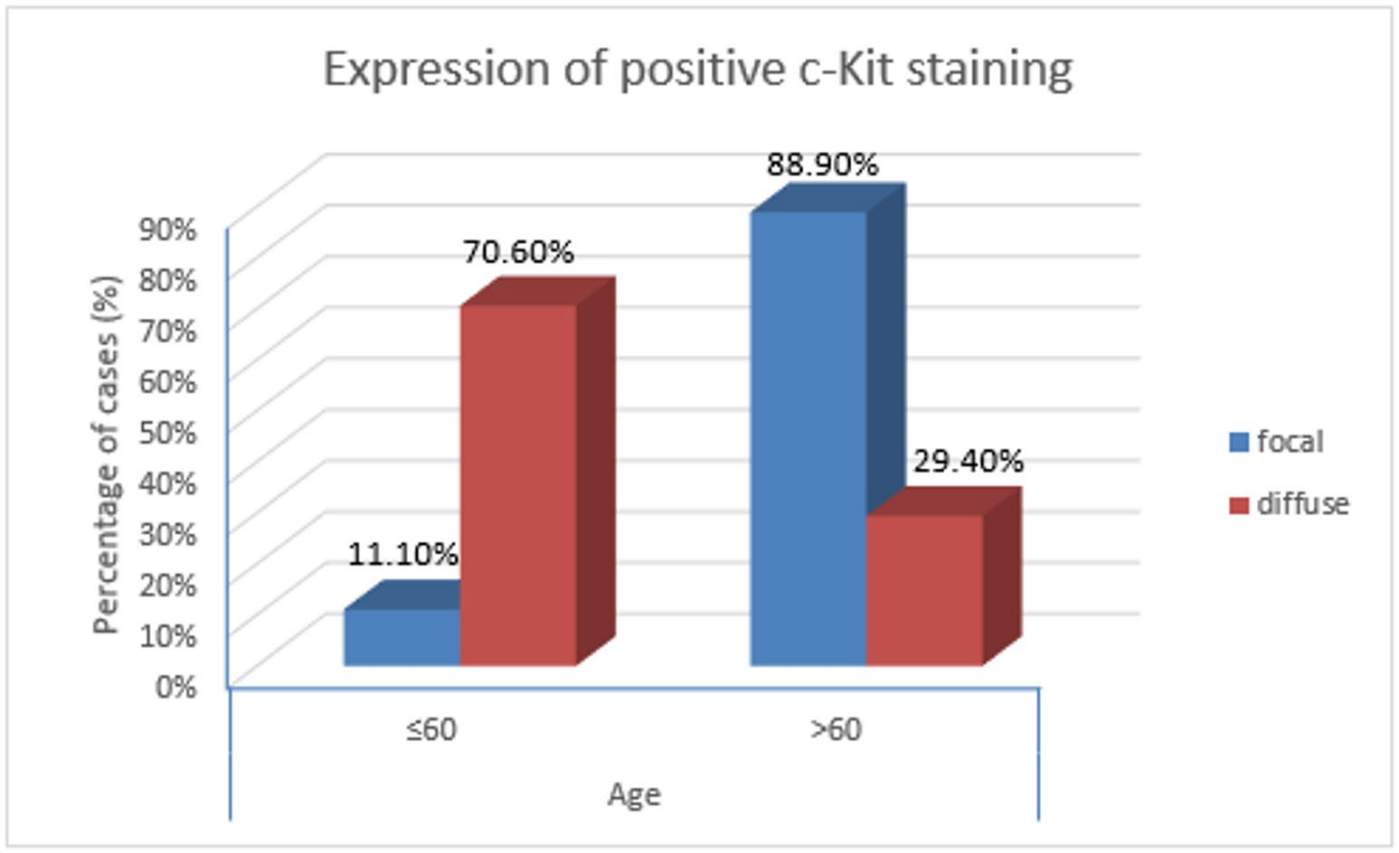
The relation between expression of positive c-Kit staining and age.

Table 2; Figure 2 demonstrate a significant correlation between focally positive c-Kit expression and age > 60 years (p-value = 0.011). Also, diffusely positive expression was significantly associated with age ≤ 60 years (p-value = 0.011). The relation between expression of c-Kit staining with histopathological variants was non-significant (p-value = 1).

**Table 3:** Relation between intensity of positive c-Kit staining and age, sex, tumor size, and histopathological variants.

		Intensity of positive c-Kit staining				p-value
		Moderate		Strong		
		Count	%	Count	%	
Age	≤ 60	1	14.3%	12	63.2%	0.073
	> 60	6	85.7%	7	36.8%	
Sex	Male	4	57.1%	9	47.4%	1
	Female	3	42.9%	10	52.6%	
Tumor Size (cm)	≤ 2	2	28.6%	15	78.9%	0.028
	> 2	5	71.4%	4	21.1%	
Histopathological variants	Low risk	6	85.7%	12	63.2%	0.375
	High risk	1	14.3%	7	36.8%	
	Total	7	100%	19	100%	

**Fig. 3:**
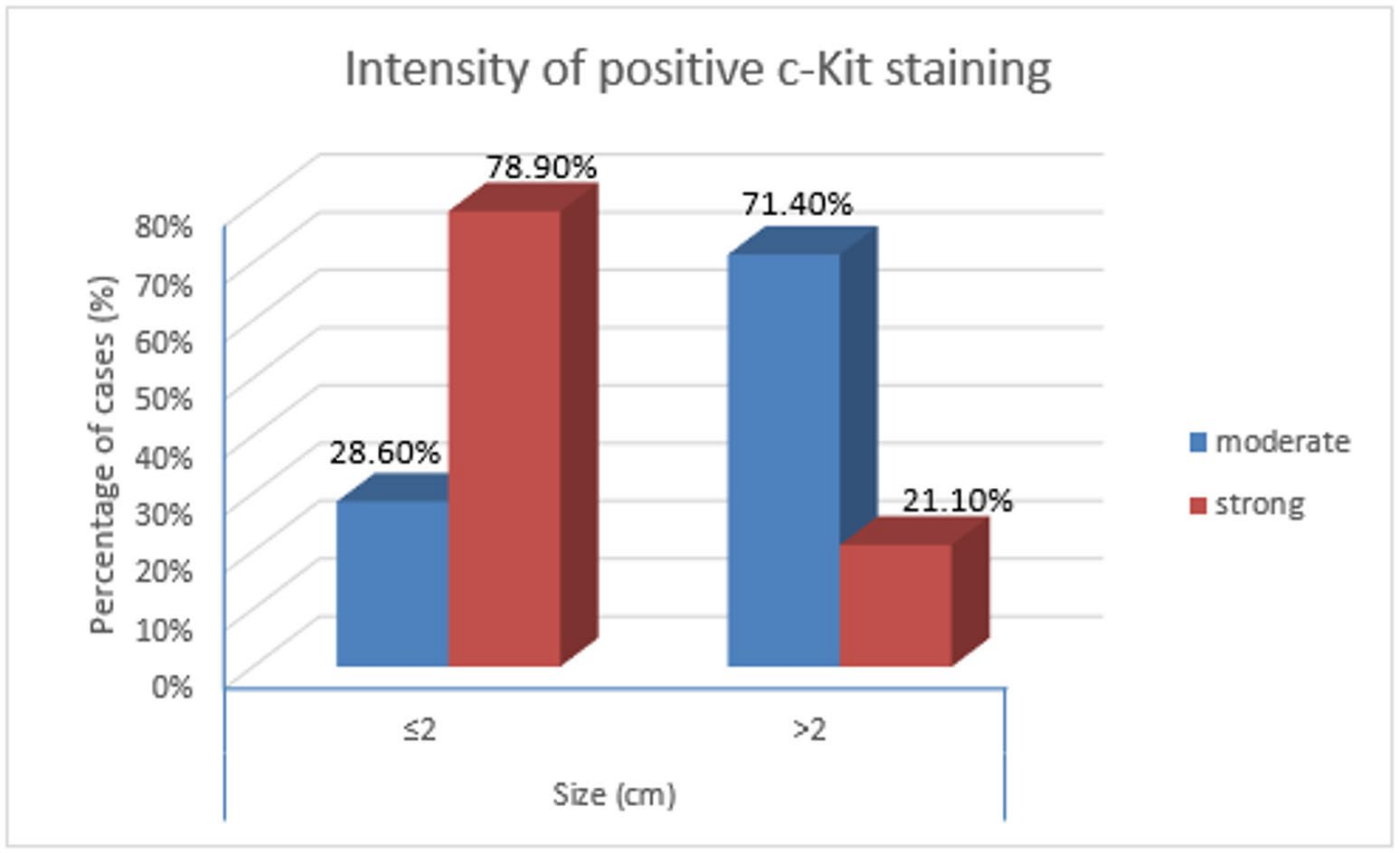
The relation between intensity of positive c-Kit staining and size.

Our study demonstrated that moderate intensity of positive c-Kit staining was statistically related to tumor size >2 cm (p-value = 0.028). Also, strong intensity was significantly associated with tumor size ≤ 2 cm (p-value = 0.028). The intensity of c-Kit staining did not significantly correlate with histopathological variants (p-value = 0.375) (Table 3; Figure 3).

**Table 4:** Clinicopathological data as well as c-Kit expression of the cSCC studied cases.

		Count	%
Histopathological diagnosis	Squamous Cell Carcinoma	21	100.0%
Age	Mean ± SD	59 ± 15.5	
	Range	35–90 years	
	≤ 60	11	52.4%
	> 60	10	47.6%
Sex	Female	7	33.3%
	Male	14	66.7%
Size (cm)	Mean ± SD	3.9 ± 3.8	
	Range	0.6–9 cm	
	≤ 2	10	47.6%
	> 2	11	52.4%
Pathological stage of cSCC	T1	8	38.1%
	T2	7	33.3%
	T3	6	28.6%
Histopathological grading of cSCC	Well Differentiated	14	66.7%
	Moderately Differentiated	6	28.6%
	Poorly Differentiated	1	4.8%
C-Kit expression	Positive	0	0%
	Negative	21	100.0%

More than half of the cSCC participants were ≤ 60 years old (52.4%) in our study. The ratio of males to females was 2:1, with males making up 66.7% of the total. Regarding tumor size, 52.4% of cases had tumors > 2 cm. In regard to the pathological stage (pT) of our cases, pT1 was slightly more predominant (38.1%) than pT2 (33.3%) and pT3 (28.6%). Concerning the histopathological grading of the studied cases, most of the cases were well differentiated (66.7%). All cSCC cases showed negative c-Kit expression (100%) (Table 4).

**Fig. 4:**
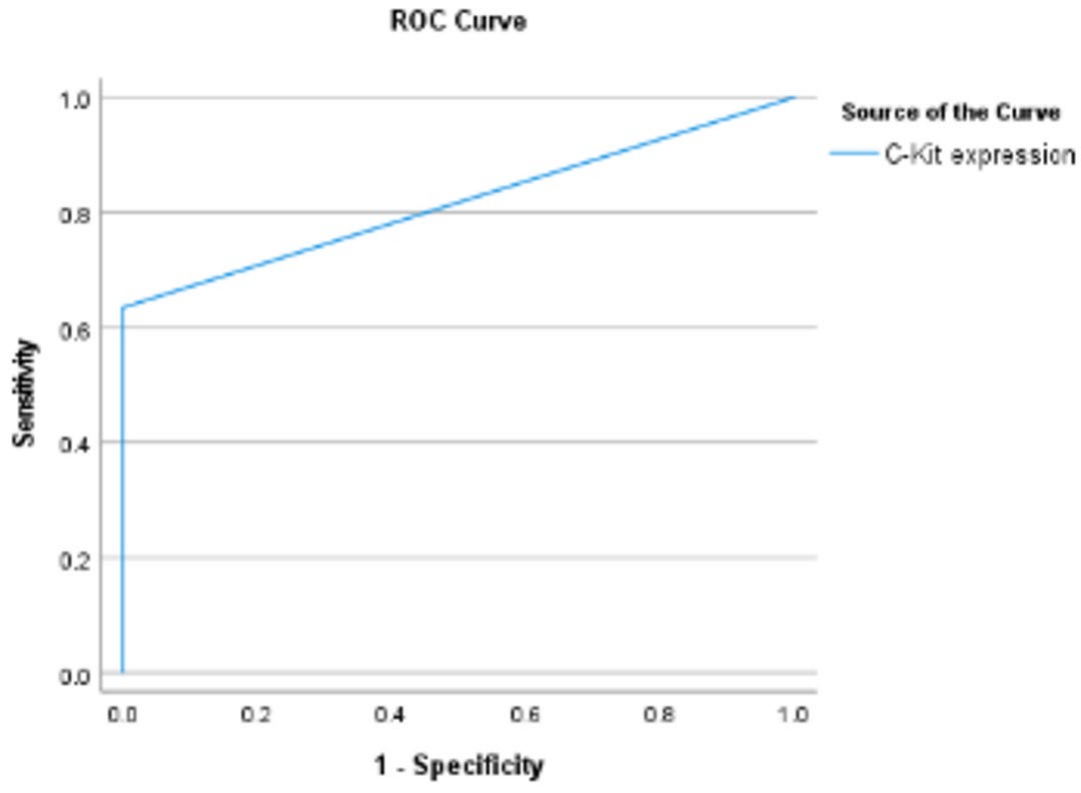
ROC curve of c-Kit expression for differentiating BCC from cSCC.

**Table 5:** Diagnostic performance of c-Kit expression for differentiating BCC from cSCC based on ROC curve analysis.

Area under curve	*p*-value	95% Confidence Interval		Sensitivity %	Specificity %
		Lower Bound	Upper Bound		
0.817	< 0.001	0.714	0.920	63.4	100

ROC curve analysis demonstrated that c-Kit expression significantly differentiated between BCC from cSCC, with an AUC of 0.817 (95% confidence interval: 0.714–0.920, p-value < 0.001), a sensitivity of 63.4% and a specificity of 100% (Figure 4; Table 5).

**Fig. 5:**
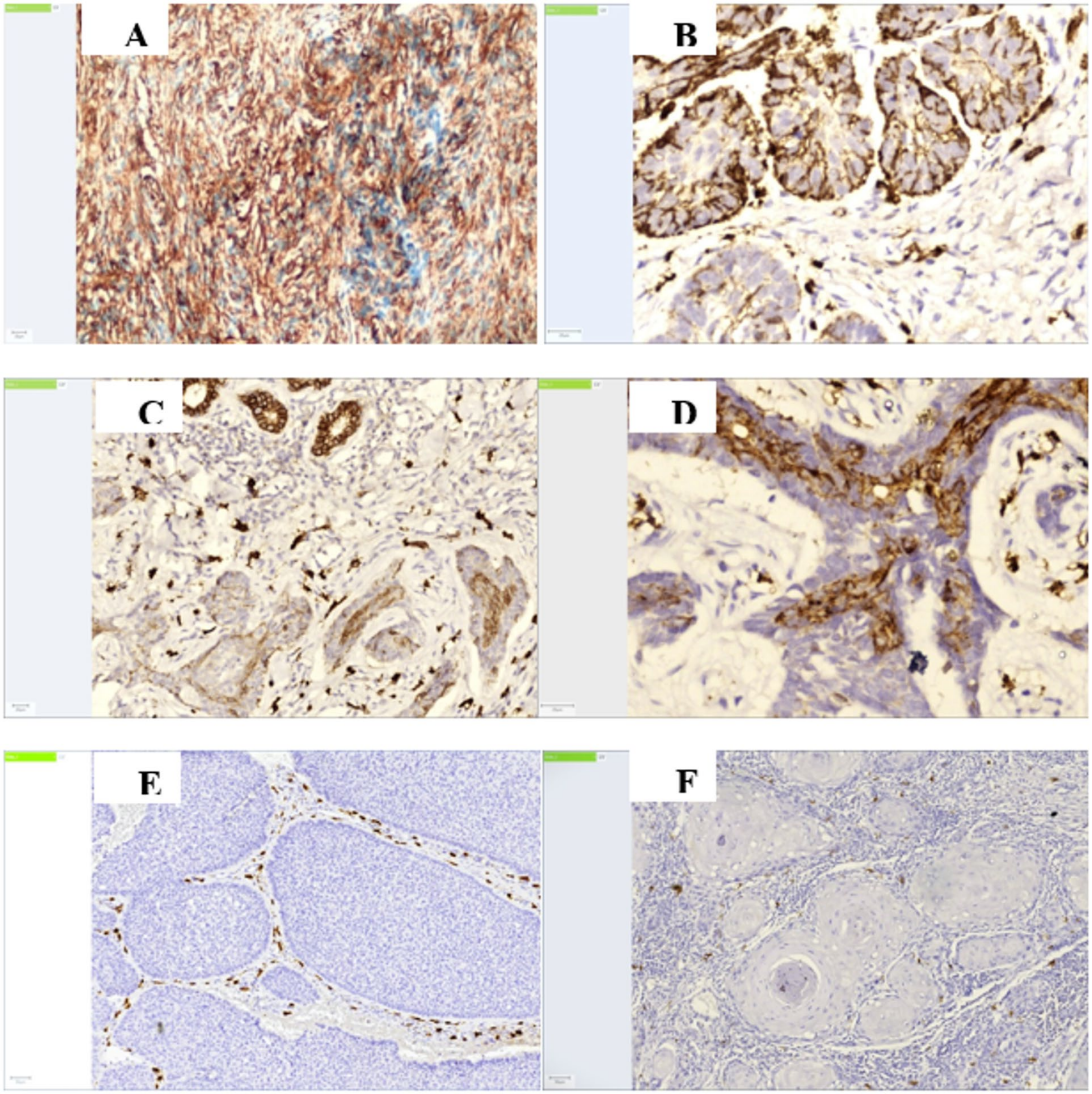
Immunohistochemical expression of c-Kit in BCC and cSCC. **A**: GIST (positive control) shows diffuse positive c-Kit expression with strong intensity (IHC, original magnification (OM) x200; scale bar = 20 μm). **B**: Nodular BCC shows diffuse positive c-Kit expression with strong intensity (IHC, OM x400; scale bar = 20 μm). **C**: Nodular BCC shows focal positive c-Kit expression with moderate intensity in comparison to the staining of normal eccrine ducts (IHC, OM x200; scale bar = 20 μm). **D**: Morpheaform BCC shows diffuse positive c-Kit expression with strong intensity (IHC, OM x400; scale bar = 20 μm). **E**: Nodular BCC shows negative c-Kit expression (IHC, OM x100; scale bar = 50 μm). **F**: Well**-**differentiated cSCC shows negative c-Kit expression (IHC, OM x100; scale bar = 50 μm).

As shown in Fig. 5, c-Kit immunohistochemical expression in BCC and cSCC was assessed.

## Discussion

In this study, 62 cases of BCC (*n* = 41) and cSCC (*n* = 21) were included.

According to this study, c-Kit expression was positive in 63.4% of BCC cases. Also, Ramezani et al.^10^ reported that the c-Kit marker was expressed in 89.6% of 29 BCC patients. However, Goto, K^14^. demonstrated only 4 out of 25 cases (16%) were positive for c-Kit expression. These variances could be attributed to differences in the number of investigated cases and histopathological variants.

According to this study, the majority of positive c-Kit expression was diffuse (65.4%), with a strong staining intensity (73.1%). Other studies showed variable results. Goto, K^14^. demonstrated 3 out of 4 cases (75%) were focal and weakly positive. Ramezani et al.^10^ illustrated 15 out of 26 cases (57.7%) were weakly positive, 11 cases (42.3%) were moderately positive, and no cases (0%) were strongly positive. Differences in the number of patients examined, histopathological variants, antibody clones utilized, immunohistochemistry technique, or positivity score criteria could all account for these discrepancies.

A significant relationship was found between focally positive c-Kit expression and age over 60 years (p-value = 0.011). A significant correlation was also found between diffusely positive expression and age ≤ 60 years (p-value = 0.011).

Positive c-Kit staining with a moderate intensity was statistically associated with tumor size >2 cm (p-value = 0.028). Additionally, strong intensity was significantly correlated with tumor size ≤ 2 cm (p-value = 0.028). To our knowledge, no other researchers have discussed these previous points to compare with our results.

This study illustrated that all cSCC cases showed negative c-Kit expression. Within the same track, Goto, K^14^. reported that 100% of 25 patients with cSCC showed negative c-Kit expression. But Goto et al.^13^ and Ramezani et al.^10^ found that five out of 40 cases (12.5%) and 6 out of 31 (19.3%) cases showed positive c-Kit expression, respectively. These differences could be explained by the variations in the number of studied cases and histopathological grades.

The ROC curve analysis demonstrated good diagnostic performance of c-Kit in differentiating BCC from cSCC, showing high specificity with moderate sensitivity. These findings suggest that c-Kit may be used in combination with routine histopathological evaluation as a complementary diagnostic marker rather than as the sole diagnostic method.

The ROC curve analysis demonstrated good diagnostic performance of c-Kit in differentiating BCC from cSCC, with an AUC of 0.817 (95% confidence interval: 0.714–0.920, p-value < 0.001), a sensitivity of 63.4%, and a specificity of 100%. The high specificity indicates that positive c-Kit expression is highly reliable in distinguishing BCC from cSCC, whereas the moderate sensitivity suggests that c-Kit should be interpreted alongside routine histopathological evaluation rather than used as a sole diagnostic marker.

## Conclusions

Our findings revealed that more than half of BCC cases (63.4%) showed positive c-Kit expression. Assessment of c-Kit expression may be beneficial in the diagnosis of BCC and in differentiating it from cSCC. However, due to its moderate sensitivity despite its high specificity, it should be used in conjunction with routine histopathological examination rather than as the sole diagnostic marker.

The detection of c-Kit positivity in BCC, despite the diagnostic nature of the research, indicates that these cases may benefit from anti-c-Kit targeted therapies.

Positive c-Kit expression was predominantly diffuse (65.4%), with a strong intensity (73.1%). There was a substantial link between focally positive c-Kit expression and age over 60 years. Focally positive c-Kit expression was statistically related to age > 60 years. Also, diffusely positive expression was significantly associated with age ≤ 60 years. All cSCC cases (100%) showed negative c-Kit expression.

To determine the therapeutic value of c-Kit inhibitors in BCC cases where c-Kit expression is positive, further, larger research is advised. Additionally, larger studies with different histopathological variants are recommended to demonstrate the relation between c-Kit expression and histopathological variants.

## Data Availability

Data availabilityAll data generated or analyzed during this study are included in this published article.

## Abbreviations

AUC: Area under the curve
BCC: Basal cell carcinoma
cSCC: Cutaneous squamous cell carcinoma
IHC: Immunohistochemistry
ROC: Receiver operating characteristic
UVR: Ultraviolet radiation
